# Occupational Nerve Injuries due to Metallic Foreign Bodies: A Case Series of Eighteen Patients

**DOI:** 10.5704/MOJ.2403.011

**Published:** 2024-03

**Authors:** P Gupta, M Jindal, S Garg, K Garg

**Affiliations:** 1 Department of Orthopaedics, Guru Gobind Singh Medical College and Hospital, Faridkot, India; 2 Department of Orthopaedics, Kalpana Chawla Govt Medical College and Hospital, Karnal, India; 3 Department of Radiotherapy, Guru Gobind Singh Medical College and Hospital, Faridkot, India; 4 Department of Anaesthesiology, Kalpana Chawla Govt Medical College and Hospital, Karnal, India

**Keywords:** foreign bodies, lathe machines and metallic chips

## Abstract

**Introduction:**

Peripheral nerve injuries (PNIs) remain an important health problem. PNIs mostly affect young men as this age group is mostly involved in road traffic accidents and other injuries at workplace. PNI can occur from foreign bodies like metal chips while working in industries using lathe machines. Among PNI’s, injuries to the ulnar nerve, the brachial plexus and the median nerve are the most frequent lesions encountered.

**Materials and methods:**

This presentation is on a series of 18 cases of nerve injuries among industrial workers located from finger level up to the arm excluding the brachial plexus due to metallic foreign bodies entering while operating lathe machines over a period of two years with patients being followed-up over a one year period.

**Results:**

Mean age in this series was 31.3 years with age range 16-40 years and all were males. Two patients had more than one nerve involvement and one patient had associated vascular injury. All the patients showed functional improvement. Most common nerve injured was median nerve. Most common site for nerve injury was forearm. Combined lesions most commonly involved the ulnar and median nerves.

**Conclusion:**

Social cost of traumatic peripheral nerve injuries is significant since it has a higher incidence in young, previously healthy, and economically active people.

## Introduction

Peripheral nerve injuries (PNIs) remain an important health problem often leading to severe motor disabilities causing a considerable decline in the patient's quality of life. PNI’s are extremely common in various upper limb injuries.

PNIs mostly affect young men as this age group is mostly involved in road traffic accidents and other injuries at work place. PNI can occur from foreign bodies like metal chips while working in industries using lathe machines. Low velocity FB injuries are usually associated with agricultural and household activities and are mostly caused by glass, needles and wooden pieces. They are generally not associated with concomitant injuries. High velocity FB injuries are associated with industrial activity, mostly due to metallic chips. These injuries occur while operating lathe machines in steel cutting industries during which metal chips are generated which due to very high speed of machine cause various soft tissue injuries including the peripheral nerves^1^.

Among PNI’s, injuries to the ulnar nerve, the brachial plexus and the median nerve are the most frequent lesions encountered^2^. Relative dearth of published clinical studies remains a major hinderance to our knowledge regarding PNIs. Galen was the first to describe the concept of the nerve but it was Paulus Aegineta in the 7th century who documented the first nerve repair and wound closure as a military surgeon^3^.

Treatment of PNI’s is a real challenge for surgeons and physicians, since the outcome after different procedures still may be insufficient. Currently surgical repair involves either direct end-to-end anastomosis or nerve grafting if the gap between the two ends is large. Re-innervation does not mean complete return of function. To attain full function, the nerve must undergo three main processes: Wallerian degeneration (the clearing process of the distal stump), axonal regeneration, and end-organ reinnervation. Failure of any of these processes can lead to poor outcome^4^. Even after a good repair or reconstruction, original well-organised hand representation will not be achieved in adults. Thus, rehabilitation programmes are started to help patient achieve maximum possible function^5^.

The present case series focuses on peripheral nerve injuries due to metallics chips generated during the operation of metal working lathes.

## Materials and Methods

We are presenting a series of 18 cases of nerve injuries among industrial workers located from finger level up to the arm excluding the brachial plexus caused by industrial foreign bodies while operating lathe machines over a period of 2 years. Our aim was removal of foreign bodies without causing further damage and repair of concomitant injuries. All the patients were in the age group of 16 to 40 years, and all were males. All patients presented with open wound with nerve involvement. Neurology was assessed and documented. Two patients also had associated tendon injury and one patient had associated arterial injury which was repaired. All the patients were symptomatic, either reporting motor (weakness) or sensory (positive or negative) symptoms or both.

Patients included in this study had partial or complete transaction of nerves, less than one year old injury and end organs were viable. Patients with spinal cord/root lesions, iatrogenic nerve injuries, obstetric brachial plexus injuries, neuropraxia and associated bony injuries were excluded.

All injuries of the ulnar nerve i.e. main trunk and all their further branches up till forearm respectively were considered together. Similar was done for radial and median nerve. Digital branches were considered separately. Surgery was performed under GA with tourniquet applied for clear vision and to reduce blood loss. Exploration and removal of foreign body under image intensifier along with end-to-end epineural repair of the damaged nerve was done in all patients.

The cut ends of the nerve were mobilised to reduce the gap and prevent tension at the site of repair. Cut ends were freshened and secured with interrupted 9-0 nylon epineurial sutures. In 2/18 patients, due to a significant gap between the two ends, cable grafting was done using sural nerve.

After the surgery, the affected limb was splinted in functional position to prevent any abnormal attitude of the affected part and also to reduce pain. Operated limb were kept elevated to prevent oedema. At three weeks post-op, passive stretching of affected joints was started to prevent stiffness and swelling. Also, for the affected joints, full range passive ROM exercises were started exercising care and caution to prevent excessive stretch on the sutured nerve. Passive and active assisted ROM exercises were started for the unaffected extremity from day one after surgery. Patients were asked to apply moisturiser or oil daily over the skin of affected area post stitch removal to prevent it from undergoing breakdown. Patients were advised to regularly inspect for any wounds or skin colour changes in the anaesthetised hand. Periodic electrodiagnostic tests were done to look for recovery. Strengthening exercises were stared in gravity eliminated plane once patient reached MMT grade 2 power and when patient reached MMT grade 3 power resisted exercises were given manually.

Rehabilitation procedures were supervised by the team of operating surgeon and physiotherapists. Judgement of return of motor power was done using medical research council scale and recovery of sensory function was assessed by using Mackinnon-Dellon scale along with Tinel sign progression.

## Results

Mean age in this series was 31.3 years with age range 16-40 years and all were males. Most common nerve injured was median nerve 8/20 (40%) either singly or in combination followed by ulnar nerve 6/20 (30%) followed by radial nerve 4/20 (20%) followed by digital nerve 2/20 (10%).

Most common site for nerve injury was forearm 11/18 (61.1%) followed by arm 5/18 (27.8%) followed by hand 2/18 (11.1%). Most commonly right upper limb was injured i.e.16/18 (88.9%). Primary repair was possible in 88.9% patients while nerve grafting was required only in (2/18) 11.1% patients. Majority of the PNIs were isolated injuries. Single peripheral nerve was involved in 15/18 (83.3%) patients while more than one peripheral nerve was injured in 3/18 (16.7%) patients. Combined lesions most commonly involved the ulnar and median nerves. One patient also had associated vascular injury. In 7/18 patients, nerve repair was done within 3 days of injury, in 5/18 patients, repair was done within 3 weeks while in 6/18 patients, timing of repair was >3 weeks. All the above data is summarised in (Table I). Motor recovery was measured using medical research council scale and to assess recovery of sensory function we used Mackinnon-Dellon scale. We also used progression of Tinel's sign as another criterion for nerve recovery after repair. All the patients showed functional improvement.

**Table I:** Distribution of patients according to nerve involvement, site of injury, side involved, type of nerve repair, number of peripheral nerves Involved and time of repair.

Nerves Involved	Ulnar nerve	Median nerve	Radial nerve	Digital nerve
No of Patients	6	8	4	2
Regions Involved	Arm	Forearm	Hand	
No of Patients	5	11	2	
Side Involved	Right		Left	
No of patients	16		2	
Type of Repair	Primary repair		Nerve Transfer	
No of Patients	16		2	
No of nerves injured	Single PNI		Multiple PNI	
No of Patients	15		3	
Time of repair	<= 3 days	3 days to 3 weeks	>= 3 weeks	
No of Patients	7	3	6	

## Discussion

**Fig 1:**
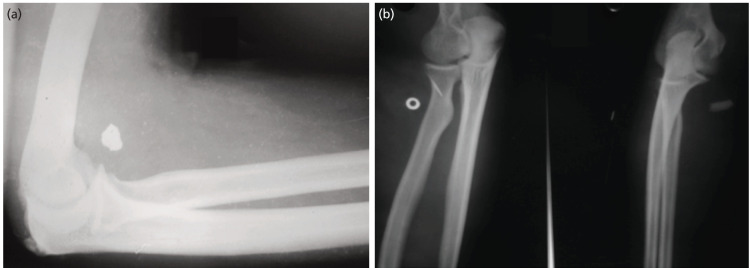
(a) A 20-year-old male with injured right proximal forearm. Foreign body seen on anterior aspect of elbow. Patient had median nerve involvement. Nerve repair was done after removal of foreign body. Patient showed significant improvement postoperatively at one year. (b) A 32-year-old male with foreign body in antero-lateral aspect of right proximal forearm. This patient had posterior interosseous nerve injury. There was partial transaction of nerve but power at MCP joint at the time of presentation was 0/5. Nerve repair was done. Power improved to 4/5 at one year post surgery.

**Fig 2:**
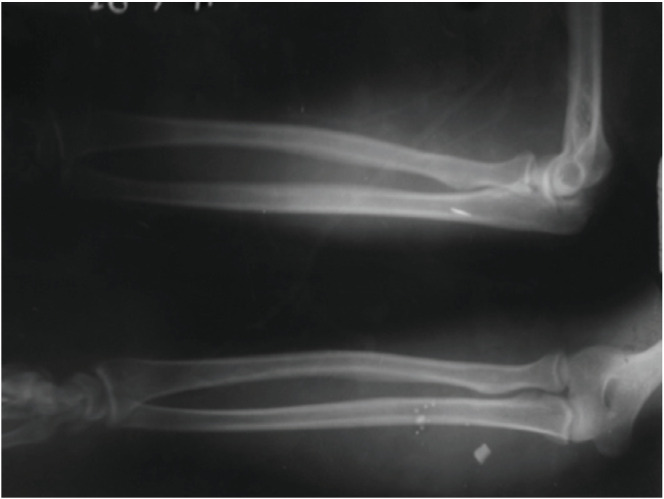
This figure shows a 25-year-old male with foreign body in medial aspect of right proximal forearm medial to ulna. This patient had complete transaction of median + ulnar nerve. Foreign body removal and nerve repair was done. There was return of power upto 3/5.

**Fig 3:**
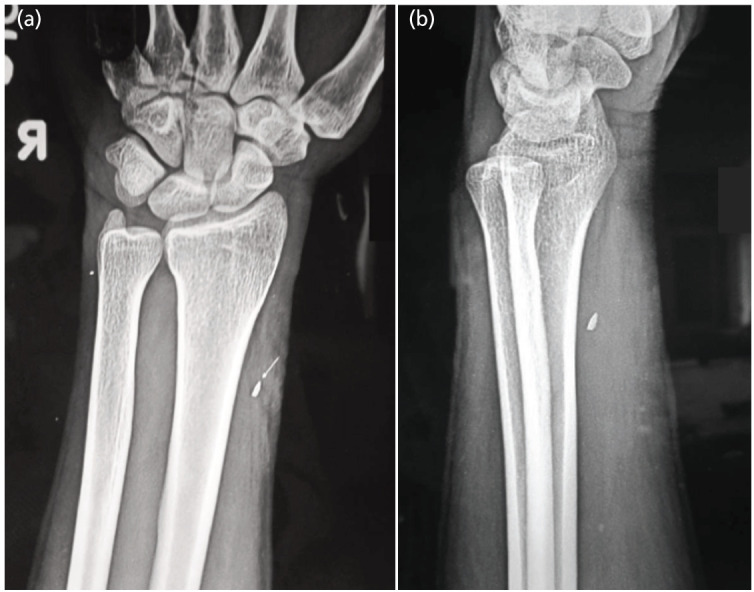
(a, b) Showing AP and Lateral views of distal forearm of 31-year-old male with foreign body in anterolateral aspect of right distal forearm. Patient had superficial radial nerve injury. Nerve repair was done. Results were excellent.

**Fig 4:**
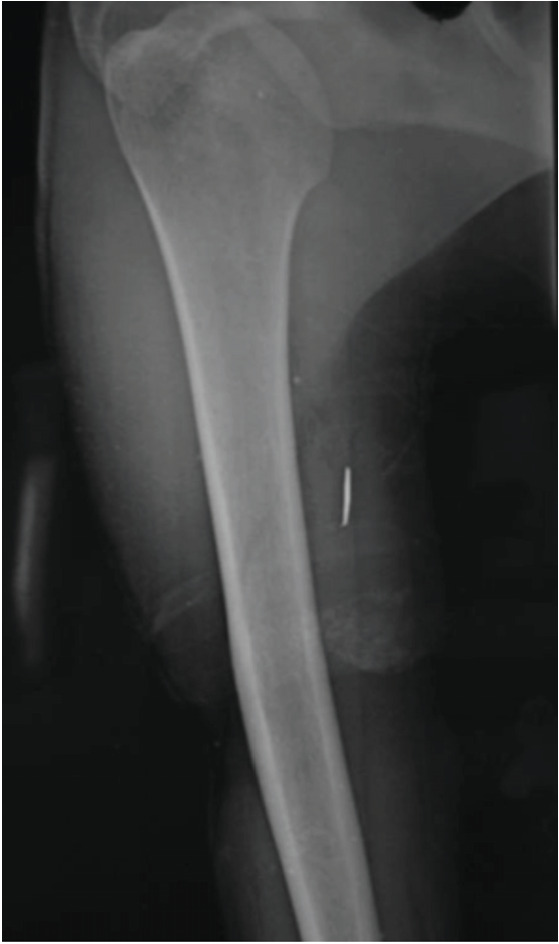
A 31-year-old male with foreign body in medial aspect right arm. Patient had median nerve+ ulnar nerve + radial nerve + brachial artery injury. Nerve and arterial repair was done. Motor recovery was only 2/5 at one year of follow-up.

**Fig 5:**
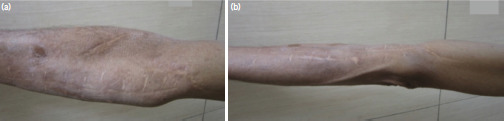
(a, b) Showing multiple scar marks on medial and lateral aspect of arm and forearm respectively indicating multiple foreign body injuries due to splinters.

Young and male patients were more likely to experience PNIs as male population is mainly involved in accidents at workplace^2^. Patient age in this series ranged from 16 to 40 years. All were males who were injured because of flying hot metal chips and coolant while operating lathe machines at their places of work. These injuries usually occurred if the machines guards or operators did not wear proper protective equipment. Injuries from these machines were more likely to occur in the upper limb. All patients in this series have injuries of upper limb. Most common site was forearm (61.1%) (Fig. 1-3) followed by arm (27.8%) (Fig. 4) followed by hand (11.1%). Most patients who had forearm injuries had entry wound on flexor aspect, whereas patients who had injuries in upper arm had entry wound on medial aspect (Fig. 5).

Previous reports indicate that the incidence is fairly common on both left and right sides^6,7^. Study conducted by Kouyoumdjian *et al* found that the right side was more affected than the left^2^. In our case series, most commonly right upper limb was injured i.e, 16/18 (88.9%) which may be because people got injured at the time of working and most people are right handed.

Most frequently affected nerves reported in literature are ulnar, radial or the digital nerves^6^. Kouyoumdjiam *et al* in his study also found that most common nerve injury is of the ulnar nerve occurring either alone or in association with median nerve^2^. In multiple nerve injuries, ulnar and median nerve lesions together are most frequent because of their close anatomical relationship, specifically in the distal forearm and wrist, probably because of the greater exposure to knife and glass wounds during performance of manual tasks^2^. In our case series however, most common nerve injured was median nerve (40%) either singly or in combination (Fig. 1, 2, 4) followed by ulnar nerve (30%) followed by radial nerve (20%) (Fig. 1, 3) followed by digital nerve (10%). A total of 2/18 patients had more than one nerve involvement. One had median and ulnar nerve involvement which was at the level of forearm while another had involvement of median, ulnar and radial nerve along with brachial artery at the level of elbow (Fig. 4).

Muscle fibre undergo atrophy as early as three weeks after denervation. However, the structural architecture of the muscle and the end-plate integrity can be maintained for up to one year^8^. In the current series, we have considered one year as the time frame for follow-up of recovery.

In patients where there is an open wound with neurological deficit, nerve exploration should always be done^5^. In our case series, all patients had open wounds at the point of entry of foreign body. So, nerve exploration was done in all patients. All patients had either partial or complete nerve transaction with complete neurological deficit. So, nerve repair was done in all patients (Fig. 6).

In study conducted by Griffin *et al*, functional recovery was better in patients where end-to-end repair was done than patients in which grafting was required^3^. In our study also, functional outcome was better where nerve grafting was not required. End to end nerve repair was done in 16/18 (88.9%) patients (Fig. 6) while nerve grafting was required in 2/18 (11.1%) patients in which cable grafting was done. Sural nerve was used as graft since it has been seen that risk for residual problems after harvesting the sural nerve is very low^9^.

Currently, there is no clear guideline regarding what should be the ideal time period after which range of motion and active mobilisation should be started. In the current series, limb was immobilised for three weeks followed by gentle passive ROM exercises for three weeks followed by active ROM.

Factors that influence functional recovery include age, duration between injury and repair, how much distal is the injury site and which nerve is involved^10^. It is considered that more distal the injury, superior is the nerve regeneration processes. According to literature, operations done early have a better outcome^11^. For cases of neurotmesis, the time to repair is more urgent, but still varies from a three day to a three-week window^12^. If the cut ends of nerve are sharp, immediate repair is done whereas if the ends are crushed with nerve in continuity, it can be left for clinical follow-up^13^. Campbell recommends repair within 72 hours for sharp transection^11^. In another study conducted by Wang *et al*, earlier repair (within the first 24 hours) did not demonstrate improved outcomes^12^. In our case series, no significant difference was found in the results based on time duration.

Two major factors favouring a good functional outcome are youth and distal injury^11^. The more distal the injury to the neuron, the more likely it is to recover^4^. In the present series, since all of our patients belonged to younger age group location of injury was more important factor. It was found that patients with injury in forearm and hand had a better outcome compared to those where site of injury was the arm. Also, patients where single nerve was injured in forearm or hand and nerve grafting was not required had the best recovery.

There is no clear standardised way to evaluate outcome that would cover all types of nerve injuries^14^. Medical research council scale and Mackinnon-Dellon scale are based on subjective findings. The lack of one authenticated scoring systems leads to inter observer variability while comparing outcome.

In the current series, motor power was 0/5 in all 18 patients pre-operatively. Post-operatively, it was seen that power recovery was 3/5 in 6/18 patients while it was 4/5 in 9/18 patients, 2/5 in 3/18 patients while there was no recovery in two patients which underwent nerve transfer at the end of one year follow-up. Three patients in which recovery was 2/5 were those where more than one nerve was injured and where site of injury was very proximal. According to Mackinnon-Dellon scale, sensory recovery was S3 in 8/18 patients, S3+ in 7/18 patients and S1 in 3/18 patients. These three patients in which sensory recovery was poor was also the same in which motor recover was poor. Progression of recovery was also assessed by looking for progression of Tinel's sign from six months post-op to one year postoperatively.

For the upper extremity, Barrios *et al* found the median nerve to have the best recovery, while Secer *et al* found the radial nerve to be the best^15,16^. But study conducted by Wang *et al* did not find any statistically significant difference amongst the peripheral nerves anatomically for motor sensory recovery^12^. In our study also, we could not find differences in recovery on the basis of which peripheral nerve was involved.

Even if the injured nerve is perfectly repaired, regeneration occurs only about 50% of neurones most probably due to sub-optimal reconstruction of fascicles^17^. All these events sometimes lead to neuroma formation. However, incidence of neuroma is low especially in the upper limb^18^. In our case series, neuroma formation was seen only in one patient who had clinical findings, such as localised pain, sensory disturbances, allodynia, and dysaesthesia. Patient was managed non-operatively with opioid, work modification and psychotherapy.

## Conclusion

Thus, social cost of traumatic peripheral nerve injuries is significant since it has a higher incidence in young, previously healthy, and economically active people. The surgeon should always anticipate more internal damage than visible on assessment, use adequate incisions, and use tourniquet if possible. Removal should not be attempted under local anaesthesia and without image intensifier in locating foreign bodies intra-operatively. If possible, surgeon should demonstrate the removed foreign body to the patient. Also, for effective public health policy, there is need of preventive education and enforcement of safety regulations for the informal occupational sector. Some limitations of this study include very small sample size.
